# Chronic Rhinosinusitis at the Interface of Type 2 Inflammation, Epithelial Barrier Dysfunction, and Microbiome Dysbiosis

**DOI:** 10.3390/microorganisms14020386

**Published:** 2026-02-06

**Authors:** Konstantinos Petalas, George N. Konstantinou

**Affiliations:** 1 Department of Allergy and Clinical Immunology, 251 General Air Force Hospital, 115 25 Athens, Greece; kpetalas@hotmail.com; 2 Department of Allergy and Clinical Immunology, 424 General Military Training Hospital, 564 29 Thessaloniki, Greece

**Keywords:** chronic rhinosinusitis, allergic rhinitis, type 2 inflammation, epithelial barrier, dysbiosis, sinonasal microbiome, *Staphylococcus aureus*, biofilm, biologics

## Abstract

Chronic rhinosinusitis (CRS) is a heterogeneous inflammatory disease of the nasal and paranasal sinus mucosa with substantial impact on quality of life. Although atopy and/or allergic rhinitis frequently coexist with CRS, often alongside type 2-skewed inflammation, the extent to which allergic mechanisms define a discrete CRS entity remains debated, in part due to inconsistent operational definitions and overlapping clinical phenotypes. In parallel, culture-independent sequencing studies have reframed CRS as a disorder of host–microbe interactions, with many cohorts reporting reduced sinonasal microbial diversity, enrichment of potentially pathogen taxa (including *Staphylococcus aureus*), and biofilm-associated community states. However, causality and directionality remain uncertain. In this narrative review, we synthesize evidence at the interface of epithelial barrier dysfunction, type 2 cytokine networks (IL-4/IL-13/IL-5), and microbiome dysbiosis, highlighting where data are consistent across studies versus where findings are heterogeneous or predominantly associative. We discuss representative allergy-associated CRS prototypes such as allergic fungal rhinosinusitis and central compartment atopic disease as clinical models to interrogate these interactions, while distinguishing them from non–IgE-mediated type 2 entities such as aspirin-exacerbated respiratory disease. We also summarize current data linking atopy to sinonasal microbial signatures and discuss emerging microbiome-directed interventions (topical probiotics, bacteriophages, and microbiota transfer concepts) alongside biologics and precision anti-inflammatory therapies. Finally, we highlight key knowledge gaps, including the limited endotype-resolved and longitudinal studies, variable allergic phenotyping in microbiome research, and the need for standardized definitions and biomarker-driven stratification to clarify clinical utility and to guide mechanism-informed therapeutic trials.

## 1. Introduction

Rhinitis is suggested by symptoms of nasal congestion, rhinorrhea (anterior or posterior nasal discharge), sneezing, and pruritus. It can be classified by mechanism as allergic or non-allergic, after excluding other conditions with overlapping symptoms [1]. Allergic rhinitis (AR) is a chronic IgE-mediated inflammation of the nasal mucosa, characterized by rapid symptom onset upon allergen exposure (e.g., pollen, dust mites, pet dander, mold spores). Sensitization to inhaled allergens is confirmed by skin prick testing or serum-specific IgE assays. Notably, some patients exhibit localized nasal IgE production without systemic atopy—a phenomenon known as local AR or “entopy” [2].

Chronic rhinosinusitis (CRS), in adults, is defined by ≥12 weeks of two or more symptoms (one of which must be nasal blockage/obstruction/congestion or nasal discharge) ± facial pain/pressure ± reduction/loss of smell, and evidence of sinus mucosal inflammation on endoscopy or CT [3]. CRS is commonly phenotyped as CRS without nasal polyps (CRSsNP) or with nasal polyps (CRSwNP) [3]. Importantly, CRS is not a uniform disease, rather, it encompasses multiple clinical phenotypes and distinct inflammatory endotypes [4].

CRS endotyping and sinonasal microbiome profiling have advanced rapidly, yet they are often discussed in parallel rather than as an integrated pathobiologic framework. This review therefore examines CRS at the interface of type 2 inflammation, epithelial barrier dysfunction, and microbiome dysbiosis, with emphasis on CRS subsets in which atopic features may be mechanistically relevant. Accordingly, the aim of this narrative review is to address three linked questions: (1) under what circumstances do atopy and allergic rhinitis plausibly contribute to CRS pathophysiology and disease expression beyond simple comorbidity; (2) how do epithelial barrier defects and type 2 programs shape host–microbe interactions and dysbiosis patterns observed in CRS; and (3) what are the most clinically actionable implications for endotype-informed management and emerging microbiome-directed strategies. In contrast to prior reviews that primarily list CRS endotypes or summarize microbiome composition studies in isolation, we synthesize mechanistic and clinical evidence across these domains, using representative type 2/allergy-associated CRS prototypes to highlight where evidence is consistent, where it remains associative, and where key knowledge gaps preclude causal inference and standardized definitions.

## 2. Epidemiology

AR is among the most prevalent chronic airway disorders, but reported rates vary substantially depending on geography and on whether definitions rely on self-reported symptoms or physician-confirmed diagnosis [5,6,7,8,9]. In large adult surveys, self-reported AR affects approximately 10–40% of individuals, whereas physician-confirmed AR prevalence is up to 14% in the United States [5,6,7]. Comparable burden is reported in other Western settings, with approximately 20% physician-diagnosed AR in Canada and 17–28.5% prevalence across European populations [8,9].

Non-allergic rhinitis (NAR) is also common (estimated at 17–52% of adults), and mixed rhinitis is reported in up to one-third of patients, complicating epidemiologic attribution of nasal symptoms to IgE-mediated disease alone [10,11,12].

Because rhinitis and acute sinonasal syndromes can mimic CRS symptom profiles, rhinitis epidemiology is an important contextual confounder when interpreting symptom-based CRS prevalence surveys and allergy–CRS comorbidity estimates [3,13]. In symptom-based population surveys, CRS prevalence ranges widely from about 5.5% to 28%, with the cited population-based estimates derived largely from Europe and North America but also from South America and East Asia (e.g., São Paulo and seven Chinese cities) [3,14,15]. Such symptom-only definitions can overestimate true CRS because similar symptoms occur in rhinitis and acute sinusitis, and symptom criteria show variable reliability compared with objective assessment [3,13]. When objective evidence of sinonasal inflammation (endoscopy or CT) is required, reported CRS prevalence is lower, typically around 3–6% in population-based studies [13,16,17].

Across epidemiologic studies, approximately one-third of CRS patients are estimated to have nasal polyps, although the proportion varies by population and polyp definition [3].

Among patients with CRS, coexistent AR is frequently reported (roughly 25–70%), but estimates vary by CRS phenotype (CRSsNP vs. CRSwNP), by recruitment setting (community-based cohorts vs. tertiary-care series), and by how allergy is defined and measured [18,19,20]. Accordingly, the broad dispersion of prevalence and comorbidity figures limits direct cross-study comparisons and helps explain why epidemiologic inferences about allergy–CRS relationships remain heterogeneous across the literature [20].

Future epidemiologic work would benefit from standardized CRS case definitions that include objective confirmation, harmonized allergic phenotyping, and broader geographic representation to improve the interpretability and generalizability of allergy–CRS associations [3,13,16,17,20].

## 3. CRS Phenotypes and Endotypes

CRS is a heterogeneous condition that can be described at two complementary levels: clinical phenotypes and inflammatory endotypes. Phenotypes are classified by observable clinical features, for example, the presence of nasal polyps (distinguishing CRSwNP vs. CRSsNP), laterality (unilateral vs. bilateral disease), or specific co-morbidities, and are primarily used for diagnosis and initial management decisions. Recognized phenotypic subsets of CRS include conditions such as aspirin-exacerbated respiratory disease (AERD, also called Samter’s triad), allergic fungal rhinosinusitis (AFRS), central compartment atopic disease (CCAD), eosinophilic granulomatosis with polyangiitis (EGPA), granulomatosis with polyangiitis, CRS with immunodeficiency, CRS with primary ciliary dyskinesia, cystic fibrosis-associated CRS, and others [3,21,22]. Table 1 summarizes key clinical triggers/associations, immunopathology/endotype features, microbiome considerations, and representative management approaches for the three archetypal type 2/allergy-associated CRS entities emphasized in this review (AFRS, CCAD, and AERD). The presence of atopy (allergic sensitization) or asthma is also frequently used to subclassify CRS phenotypes [23,24,25]. However, phenotype alone does not capture the myriad underlying inflammatory mechanisms, while patients with similar clinical phenotypes can have different immunopathologic drivers.

To address this, CRS can be further characterized by endotypes, which are defined by distinct pathophysiological mechanisms identified via specific biomarkers [26,27]. Endotyping relies on analyzing tissue or fluid biomarkers (cytokine profiles, cellular infiltrates, immunoglobulin levels, etc.) that reflect the dominant immune pathways in a patient [28,29]. Common sources for biomarker analysis include sinus mucosa, nasal polyp tissue, or blood samples [28,29]. An international landmark study first applied cluster analysis to biomarker profiles in CRS patients, identifying 10 potential CRS endotypes based on underlying immune signatures [30]. Four clusters were characterized by low or undetectable type 2 (T2) markers (IL-5, IgE, eosinophilic cationic protein), corresponding mostly to a non-eosinophilic, IL-5–negative endotype often seen in CRSsNP and associated with low asthma prevalence [30]. The other six clusters had high IL-5 and IgE (“T2–high” endotypes). Among these, one subgroup had moderate IL-5 with mixed CRSsNP/CRSwNP and increased asthma, while the remaining showed very high IL-5 levels and were almost exclusively CRSwNP with markedly increased asthma prevalence [30]. Notably, the clusters with highest IL-5 and IgE levels were distinguished by the presence of *Staphylococcus aureus* (*S. aureus*) enterotoxin-specific IgE in all samples [30], linking colonization by *S. aureus* (and its superantigenic toxins) to the most severe T2 endotype. This important study established clear links between endotypes and clinical features (nasal polyps, comorbid asthma) and motivated efforts to identify clinically useful biomarkers. The 10 endotype clusters were later simplified into three groups based on IL-5 levels (high IL-5, low IL-5, or essentially absent IL-5 inflammation), supporting the clinical utility of a limited marker set for stratification [31].

In practice, CRS endotypes are often dichotomized into T2 or T2-high inflammation versus non–T2 or T2-low inflammation. Interestingly, either endotype can occur within the same clinical phenotype, including CRSwNP.

**Figure 1:**
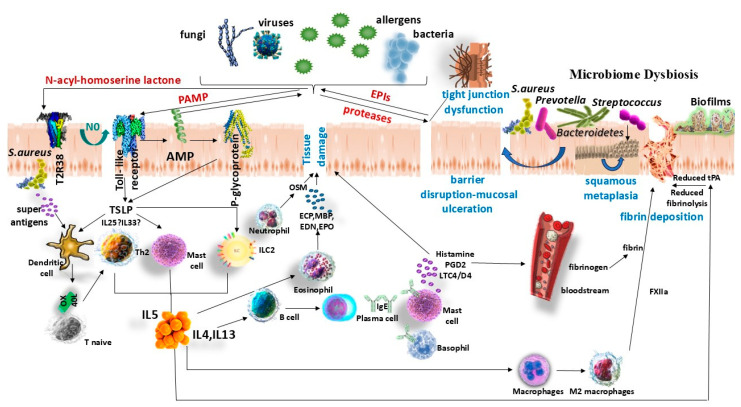
Conceptual schematic of the self-reinforcing loops linking sinonasal microbiome dysbiosis, epithelial barrier dysfunction, and type 2 (T2) inflammation in “allergic CRS”. Environmental allergens and microbes (bacteria, fungi, viruses) provide PAMPs, proteases, and superantigens that engage epithelial innate sensing pathways (e.g., TLRs) and taste-receptor signaling (TAS2R38/T2R38), modulating antimicrobial effector functions (AMPs, nitric oxide) and epithelial alarmin release (TSLP, IL-25, IL-33). Barrier injury (tight-junction dysfunction, mucosal disruption/ulceration) and impaired mucociliary clearance promote dysbiosis and biofilm formation (e.g., enrichment of *Staphylococcus aureus*, *Streptococcus*, and *Prevotella*/Bacteroidetes), which amplifies Th2/ILC2 activation, IgE class switching, eosinophilia, and mast cell/basophil mediator release, further sustaining tissue damage and permeability. Additional host pathways implicated in polypoid/T2-high remodeling include neutrophil-derived oncostatin M, epithelial P-glycoprotein (ABCB1), and coagulation/fibrinolysis imbalance (reduced tPA and excessive fibrin deposition). Abbreviations: AMP, antimicrobial peptides; ECP, eosinophil cationic protein; EDN, eosinophil-derived neurotoxin; EPO, eosinophil peroxidase; EPIs, epithelial protease inhibitors; IgE, immunoglobulin E; ILC2, group 2 innate lymphoid cells; IL, interleukin; LTC4/D4, cysteinyl leukotrienes C4/D4; MBP, major basic protein; NO, nitric oxide; OSM, oncostatin M; OX40L, OX40 ligand; PAMP, pathogen-associated molecular patterns; PGD2, prostaglandin D2; PRR, pattern-recognition receptor; tPA, tissue plasminogen activator; TLR, Toll-like receptor; TSLP, thymic stromal lymphopoietin.

Type 2 inflammatory CRS is characterized by involvement of Th2-biased immune cells: elevated Th2 lymphocytes and T2 innate lymphoid cells (ILC2s), plasma B cells, alternatively activated (M2) macrophages, dendritic cells, mast cells, and abundant eosinophils and basophils in tissues [32]. Type 2 CRS features high levels of the cytokines IL-4, IL-5, IL-13 and often elevated IgE. IL-5 is crucial for eosinophil maturation, activation, and survival, while IL-4 and IL-13 mediate goblet cell hyperplasia (mucus production), smooth muscle contraction, vascular permeability, and recruitment of inflammatory leukocytes [33]. A key upstream driver of T2 inflammation is the injured sinonasal epithelium: epithelial cells release so-called “alarmin” cytokines such as thymic stromal lymphopoietin (TSLP), IL-33, and IL-25, which activate ILC2s and prime Th2 responses [32]. This epithelial-initiated cascade is thought to underlie the robust T2 inflammation seen in polypoid CRS (Figure 1).

By contrast, non–T2 CRS can be subdivided into endotypes dominated by Type 1 or Type 3 inflammation (sometimes termed Th1- and Th17-skewed endotypes) [34,35,36]. Type 1 inflammation is marked by high interferon-γ (IFN-γ) production from Th1 cells, natural killer cells, and ILC1s [37]. Th1-driven responses activate macrophages and can induce epithelial apoptosis and neutrophil activation [38]. Clinically, type 1 inflammation has been linked to disease chronicity and is more common in CRSsNP phenotypes [39]. Type 3 inflammation, on the other hand, is driven by IL-17 and IL-22 production from Th17 cells and ILC3s, leading to neutrophil recruitment and activation [40,41]. Th17 pathways can stimulate epithelial cells to produce proinflammatory cytokines like osteopontin, which in turn promote dendritic cell maturation and further Th17 differentiation. These type 1 and type 3 endotypes often manifest with neutrophilic tissue infiltrates and are typically less responsive to corticosteroid therapy than eosinophilic T2 disease.

Beyond immunologic endotyping, CRS phenotypes also tend to cluster based on microbial community composition. Culture-independent profiling indicates that CRS subtypes can be distinguished as distinct microbial community states and that diversity metrics may relate to clinical progression and surgical outcomes [42,43] (Figure 1).

Beyond the binary clinical distinction between CRS with and without nasal polyps (CRSwNP vs. CRSsNP), contemporary frameworks also classify CRS as primary versus secondary and localized versus diffuse disease. Endotyping (e.g., type 2-high vs. type 2-low/non-type 2) cuts across these clinical phenotypes and varies by geography and comorbidity burden [44]. Throughout this narrative review, we use “phenotype” to denote clinical prototypes (e.g., AFRS, CCAD and AERD) and “endotype” to denote biomarker-defined immune patterns (e.g., T2-high, type 1, or type 3)

## 4. Definitions and Conceptual Framework

The contribution of allergic inflammation to CRS pathophysiology remains incompletely understood and at times controversial, as studies have reported conflicting results regarding the correlation of atopy with CRS severity. A key point is to distinguish between atopy and clinical allergy. Atopy refers to the state of having IgE antibodies to common aeroallergens (detectable by skin prick or in vitro tests), whereas AR is the symptomatic condition that results from IgE-mediated inflammation upon allergen exposure. Atopy is very common (roughly 50% prevalence of sensitization in the general population) [45,46], meaning many individuals are sensitized without overt symptoms. Thus, a high proportion of people (including CRS patients) may have positive allergy tests (atopy) even if nasal allergies are not clinically active.

Allergic responses, such as AR, predominantly involve a T2 immune mechanism. In an atopic individual, inhaled allergens are captured by nasal antigen-presenting cells (dendritic cells, etc.), which then activate allergen-specific Th2 cells and downstream effector cells (B cells, mast cells, eosinophils, etc.). This classical Th2 pathway underlies AR symptoms [46,47]. Consequently, CRS patients with a non–T2 endotype (e.g., neutrophil-dominant CRS) are less likely to have comorbid AR, whereas those with a T2 endotype often have concurrent AR or atopic features.

Importantly, type 2 (T2) inflammation and clinical allergy overlap but are not synonymous. T2-skewed sinonasal inflammation can be sustained by epithelial “alarmins” (TSLP, IL-25, IL-33), microbial products (including *S. aureus* enterotoxins), and other non-allergen triggers, even in the absence of demonstrable systemic atopy. Conversely, allergic sensitization is common in the general population and may coexist with CRS without necessarily being the dominant driver of sinus disease.

Within this review, we use the term “allergic CRS” pragmatically as a heuristic working descriptor (a pragmatic umbrella term rather than a formally validated endotype) to describe CRS coexistence with evidence of IgE-mediated allergy (clinically relevant allergic rhinitis or objective aeroallergen sensitization) together with sinonasal features consistent with a T2-skewed inflammation (eosinophilia, elevated IgE/IL-5, and/or polypoid remodeling), recognizing that these features increase plausibility but do not by themselves establish causality. This construct is most directly illustrated by phenotypic prototypes with strong atopic/T2 signatures, such as AFRS and CCAD. We explicitly distinguish this from AERD, a prototypic T2-high CRS entity that reflects a non-IgE drug hypersensitivity driven by dysregulated arachidonic acid metabolism, rather than classical allergen-specific IgE responses [48].

Conceptually, the sinonasal epithelium and its associated microbiota form a dynamic “niche” that integrates environmental exposures with mucosal immunity. Barrier integrity, mucociliary clearance, and epithelial innate immune sensing influence microbial community compositions, while microbial dysbiosis and biofilms can disrupt barrier function and increase epithelial cytokine release, leading to ongoing inflammation. This host–microbe feedback model provides a unifying framework to interpret how allergic/atopic inflammation may converge with microbiome dysbiosis in CRS.

## 5. The Sinonasal Microbiome and Dysbiosis in CRS

### 5.1. The Bacteriome: Diversity Loss, Pathogen Enrichment, and Community States

One prominent finding in CRS is an imbalance in the sinonasal microbiota. Distinct microbial community states have been described in CRS, each dominated by specific taxa and linked to divergent mucosal immune profiles [43]. Microbiome dysbiosis, a disruption of the normal microbial ecosystem, has been identified as a hallmark of CRS in many studies [42,43,49]. Healthy sinuses host a diverse microbiome, including commensal bacteria like *Staphylococcus epidermidis* and *Corynebacterium* species, whereas CRS patients often show an overgrowth of certain pathogenic bacteria, such as *S. aureus*, along with a decrease in overall microbial diversity. This pattern has been repeatedly observed in culture-independent sequencing studies, but it should be interpreted primarily as an association rather than proof that dysbiosis is a primary cause of CRS [42,49,50]. This shift in community structure has been associated with inflammatory patterns in CRS cohorts, but directionality remains uncertain. Increased *S. aureus* density, for example, has been associated with, and has been hypothesized to contribute to both Th1 and Th2 inflammation in nasal mucosa, exacerbating CRS and promoting polyp formation [51]. Microbial products like *S. aureus* enterotoxins can act as superantigens, and have been proposed to promote polyclonal IgE production and T2 inflammation in susceptible hosts. On the other hand, a rich and balanced microbiota might help maintain immune homeostasis. Conversely, loss of microbial diversity can skew local immunity. Interestingly, systemic microbiome links have been noted as well—for instance, decreased gut bacterial diversity has been associated with a shift toward T2 immune responses and greater propensity for atopic diseases (AR, asthma) [52]. More recently, a two-sample Mendelian randomization analysis suggested a possibly causal direction whereby CRS could influence the relative abundance of specific gut taxa (e.g., decreased *Haemophilus parainfluenzae* and increased *Bilophila*) and associated metabolic pathways [53]. These findings suggest that microbial disturbances both locally (in the nose) and distally (gut-lung axis) can shift the immune balance toward allergy and chronic inflammation in the airways.

In CRSwNP, several studies report dysbacteriosis and reduced commensal abundance with shifts in genera such as *Moraxella*, *Staphylococcus*, and *Corynebacterium*, although findings vary by geography, sampling site, medication exposure, and sequencing pipelines [54,55]. Importantly, lower diversity and specific microbial signatures have been associated with worse postoperative outcomes in some cohorts, suggesting that microbial ecology may contribute to treatment response heterogeneity [42]. Key sources of inter-study variability are summarized in Section 5.3.

### 5.2. Beyond Bacteria: Mycobiome, Virome, and Phage Considerations

Although most studies focus on bacteria (the bacteriome), the sinonasal ecosystem also includes fungi and viruses. Multi-kingdom approaches (bacteriome, mycobiome, virome) remain limited, but may be particularly relevant to allergy-associated entities such as AFRS and to defining clinically meaningful dysbiosis patterns across the united airway [55,56,57,58,59]. Recent full-length 16S and fungal ITS sequencing in AFRS characterized a low-diversity, dysbiotic bacterial community dominated by *S. aureus* (with enrichment of *Streptococcus pneumoniae* and *Haemophilus influenzae*) alongside a mycobiome enriched in *Malassezia*, *Aspergillus* and *Curvularia*, suggesting potential cross-kingdom mechanisms [56]. Paired bacteriome/mycobiome analyses coupled to cytokine profiling further indicate that microbial biodiversity can associate differently with T2 versus non-T2 inflammation in CRS, highlighting the value of multi-kingdom and endotype-resolved study designs [58]. In addition, viral detection studies and emerging virome-focused syntheses suggest that respiratory viruses may be detectable more frequently in CRS than in controls, but their causal role and their interaction with bacterial dysbiosis remain uncertain [60].

### 5.3. Methodological Heterogeneity and Key Confounders in CRS Microbiome Studies

Early work directly comparing conventional culture with 16S rRNA gene sequencing demonstrated that culture captures only a subset of the taxa identified by sequencing and that CRS cases show altered community composition with higher *S. aureus* abundance; importantly, measured diversity correlated with recent antibiotic exposure and asthma comorbidity [61]. Syntheses of the expanding literature (systematic review and meta-analysis) likewise support a dysbiosis (“community collapse”) framework while underscoring substantial inter-study heterogeneity and the need for standardized sampling and phenotyping [62,63]. Research indicates that variability between subjects is greater than the differences within a single subject’s anatomy. This supports the practical approach of using middle meatus sampling as a substitute for deeper sinus sites in many clinical studies [64].

Across studies, sinonasal microbial profiles are sensitive to recent antibiotic exposure, intranasal and systemic corticosteroid use, sampling site and technique (swab vs. tissue; middle meatus vs. sinus cavity), sequencing platform/pipeline, and host/environmental context (geography, smoking, asthma, and atopy). These factors can obscure disease-specific signals and likely contribute to the heterogeneity of reported “CRS microbiomes”, underscoring the need for standardized protocols and deeply phenotyped, longitudinal cohorts [55,62,63,64].

## 6. Mechanistic Links: Allergic CRS at the Microbiome–Barrier–Type 2 Inflammation Interface

CRS is a multifactorial inflammatory syndrome resulting from a complex interplay of environmental factors and host immune responses. Key elements implicated in CRS pathogenesis include: epithelial barrier dysfunction, microbiome dysbiosis, and dysregulated immune/inflammatory pathways (varying by endotype and geography). Recent data also suggest roles for epithelial-derived exosomes and neural interactions, but these remain under investigation. In simple terms, an abnormal sinus epithelial barrier and changed microbial community can trigger an excessive, chronic immune response in susceptible individuals.

### 6.1. Clinical and Epidemiologic Evidence Linking Allergy and CRS

The literature on allergy’s impact in CRS shows mixed findings. An evidence-based review of the association between atopy and CRS found variable results. In CRSsNP, 4 of 9 studies reviewed showed an association with allergy, while 5 did not. In CRSwNP, 10 of 18 studies found a positive association with allergy, 7 found no relationship, and 1 was equivocal [20]. In one study, Li et al. specifically examined atopic status (sensitization without necessarily clinically relevant symptoms) in CRSwNP patients and found no correlation between atopy (total IgE levels, eosinophil cationic protein, or multiplex allergen screening) and CRS disease severity or recurrence risk [65]. Similarly, clinical outcomes like symptom scores, endoscopic grading, and CT scores did not differ significantly between atopic and non-atopic CRSwNP in that study. These findings suggest that simply having allergen-specific IgE, regardless of its clinical significance, may not predict how severe or resistant a person’s CRS is.

Separate experiments have examined mechanistic links between nasal allergy and sinus inflammation. In a classic study, Adkins et al. instilled radiolabeled allergen into allergic volunteers and found that the allergen was mostly confined to the nasal cavity and oropharynx, failing to penetrate into the paranasal sinuses [66]. This suggests a physical anatomic barrier: inhaled allergens do not readily reach sinus mucosa in significant quantities, which could explain why nasal allergy might not directly inflame the sinuses. On the other hand, a study by Hamizan et al. identified specific endoscopic signs in the nose that correlate strongly with inhalant allergy. They found that diffuse middle turbinate edema and polypoid changes in the central nasal compartment had an excellent positive predictive value for systemic inhalant allergy (though sensitivity was low) [67]. In other words, allergic patients tended to show edema of the middle turbinate on endoscopy, a feature that can hint at underlying atopy in CRS patients.

Large epidemiologic studies likewise support an association between allergy and CRS in certain populations. A recent UK study of 1470 patients reported that CRS patients were significantly more likely than controls to have confirmed inhalant allergies (by skin prick or RAST) [68]. Interestingly, among CRS subgroups in that study, CRSsNP patients had a higher rate of allergy than CRSwNP (31% vs. 20% with confirmed inhalant allergy), largely due to greater dust mite sensitization in the CRSsNP group (16% vs. 9%) [68]. Another study from a tertiary care center found atopy in 52% of CRSsNP patients and 76% of CRSwNP patients. Notably, in the CRSsNP subset, atopic patients had more severe sinus CT scores than non-atopic patients [69]. These data indicate that although allergy is common in CRS (particularly among polyp patients) its presence alone does not always determine severity. However, in certain groups like CRSsNP, atopy may contribute to increased radiographic disease burden.

Collectively, the epidemiologic association between atopy and/or AR and CRS is heterogeneous and does not support a universal causal model across all CRS phenotypes [3,20,44]. Reported differences likely reflect variation in CRS case definitions (symptom-based vs. objective endoscopic or radiographic) and in allergy phenotyping (sensitization vs. clinically active AR), which can lead to misclassification and dilution of true signals [3,13,20,45,46,47]. Most studies are cross-sectional, limiting temporal inference and permitting reverse causation or shared-risk-factor confounding [20,68,69]. Referral bias in tertiary-care cohorts may inflate comorbidity estimates and severity correlations compared with population-based samples [20,69]. Effect modification by endotype and comorbid asthma (including T2-high prototypes such as AERD/AFRS/CCAD) likely explains why associations appear stronger in selected subgroups than in unselected CRS populations [25,44,48,70,71,72,73]. Clinically, these data support selective allergy evaluation when suggested by history or endoscopic patterns (e.g., central compartment changes), while emphasizing that sensitization alone is not a reliable marker of CRS severity or recurrence [20,25,65,67].

Overall, T2-high CRS (eosinophilic, polypoid disease) tends to coexist with other atopic disorders. Patients with CRS driven by T2 inflammation often have a history of AR and/or asthma. In fact, AR appears to play a particularly important role in certain CRS subtypes that are characterized by pronounced T2 immunity. Notably, AERD, AFRS, and the more recently described CCAD [71,72,73]. These subtypes could be considered examples of “allergic CRS” phenotypes, as atopic or allergic mechanisms are central to their pathogenesis. Some experts have suggested the term “allergic CRS” to describe a distinct CRS phenotype characterized by a Th2-skewed endotype in atopic individuals [20], although this concept overlaps with the categories mentioned above.

### 6.2. Atopy-Associated Microbial Signatures and T2-High Dysbiosis

Microbiome dysbiosis may represent a mechanistic link between atopy and CRS persistence. In a CRS cohort incorporating allergic phenotyping, concurrent AR was associated with differences in the relative abundance of sinonasal bacteria and predicted functional pathways (including lower *Corynebacterium* spp. abundance in allergic vs. non-allergic CRS) [74]. Comparative studies involving allergic rhinitis, CRS, and healthy controls similarly show differences at the group level in both diversity and taxonomic composition [75,76]. Importantly, most CRS microbiome studies do not explicitly define “allergic CRS” as a distinct entity. Instead, atopy is often noted as a comorbidity or covariate, which limits the ability to determine causality. A systematic, standardized approach to allergic phenotyping (including history, skin testing/specific IgE, and where possible, local IgE) along with microbial profiling, remains an unmet need for identifying microbiome signatures associated with allergic endotypes that could be targeted for intervention.

Cross-sectional studies comparing allergic rhinitis, CRS, and healthy controls also find differences in microbial diversity and composition between groups, supporting the idea that T2 inflammation can alter the sinonasal environment [75,76].

Within T2–high CRS, eosinophilic CRSwNP has been associated with characteristic microbial alterations and correlations with eosinophilic inflammation and innate lymphoid cell signatures [50,77,78]. These observations support the hypothesis that dysbiosis can enhance T2 circuits via epithelial alarmins and antigenic stimulation, such as exposure to *S. aureus* enterotoxins and local IgE production in susceptible hosts [51] (Table 2). In a steroid-free cohort of NSAID-exacerbated respiratory disease (N-ERD), increased staphylococci and reduced corynebacteria correlated with IL-5 and other T2 markers, supporting a microbiome-T2 linkage while minimizing corticosteroid confounding [79].

The available evidence linking atopy/AR with sinonasal microbial signatures in CRS is dominated by cross-sectional observational studies, with limited longitudinal or interventional data, which constrains inference about directionality and causality [55,62,63,64,74,75,76]. Accordingly, reported “atopy-associated” microbial patterns should be interpreted as associations that may reflect confounding by medication exposure, sampling site, and underlying CRS endotype heterogeneity [55,62,63,64].

### 6.3. Epithelial Barrier Dysfunction as a Driver of a Pro-Dysbiotic, Type 2-Skewing Microenvironment

The sinonasal epithelium is the first line of defense and is often structurally and functionally abnormal in CRS. Normally, respiratory epithelial cells form tight junctions, creating a physical barrier that prevents the entry of allergens, microbes, and pollutants. They also facilitate mucociliary clearance, in which a layer of mucus traps inhaled particles, and coordinated ciliary beating moves the mucus out of the sinuses. In CRS, this barrier function is compromised. Epithelial cells from CRS patients show reduced integrity of tight junction proteins and impaired ciliary function. They are also frequently in a persistently “activated” state, secreting an excess of cytokines. Under healthy conditions, the epithelium actively participates in immune surveillance. It secretes antimicrobial peptides (such as defensins and lactoferrin) and produces cytokines/chemokines that recruit immune cells to clear pathogens [80]. If the epithelial barrier is compromised or dysfunctional, as seen in CRS, external agents (allergens, bacteria, fungi, pollutants) can more readily penetrate and persist on the mucosal surface, thereby maintaining an ongoing inflammatory response. Notably, epithelial barrier defects are implicated in other allergic diseases (atopic dermatitis, asthma, etc.) [81], which parallels observations in CRS and AR.

When inhaled pathogens or irritants contact the epithelium, they interact with different pattern-recognition receptors (PRRs) located on the cell surface or within the epithelium. Distinct PRRs (such as Toll-like receptors) recognize microbial structures and initiate signaling cascades. In the context of CRS and AR, activating airway epithelial PRRs results in the release of pro-inflammatory and T2-skewing cytokines, particularly TSLP, IL-25, and IL-33 [80]. These epithelial-derived alarmins are key triggers for downstream allergic inflammation. They condition mucosal dendritic cells to drive Th2 differentiation and directly activate ILC2 cells to produce IL-5 and IL-13. Therefore, exposure to certain microbes or pollutants can cause epithelial cells to effectively activate toward a Th2 immune response in susceptible individuals. Elevated levels of TSLP, IL-33, and IL-25 have been found in polyp tissues and are believed to sustain the chronic T2 environment in CRS.

Another consequence of epithelial dysfunction in CRS is impaired mucociliary clearance, which further aggravates microbial dysbiosis. Structural or functional ciliary defects, whether due to genetic conditions (such as primary ciliary dyskinesia) or acquired inflammation, result in accumulation of retained mucus within the sinuses. This stagnant mucus creates an environment that promotes bacterial growth and biofilm formation. Indeed, biofilms (complex communities of bacteria embedded in a protective matrix) are commonly identified on the sinus mucosa in CRS patients and are associated with recalcitrant disease. Organisms within biofilms (e.g., *Pseudomonas*, *Staphylococcus*) are more resistant to antibiotics and immune clearance [82,83]. Chronic rhinitis patients, including AR patients, have been noted to have prolonged mucociliary transit times as well [84], indicating that allergic inflammation itself can slow mucociliary clearance. This sets up a clinically plausible feedback loop in which inflammation can impair clearance and favor microbial persistence, while microbial persistence can further amplify inflammation. However, the directionality and relative contribution of each step likely varies by endotype and remains incompletely resolved in human studies [55,62,63,64,82,83,84].

As the epithelial barrier becomes more permeable, allergens and microbes gain easier access to submucosal immune cells, leading to persistent immune activation. In both CRS and AR, a leaky epithelium means greater allergen uptake and presentation, fueling the Th2 allergic response [84]. There is also evidence that chronic inflammation in CRS alters the behavior of basal epithelial progenitor cells (the stem cells of the epithelium), skewing their differentiation and perhaps preventing proper barrier restoration [85]. These factors combine to sustain inflammation even in the absence of acute infections.

Once the T2 inflammatory cascade begins, a feedback loop occurs. ILC2s and Th2 cells produce the signature cytokines IL-5, IL-13, and IL-4, which further amplify the response (IL-5: eosinophil maturation in the bone marrow, mobilization to the blood, survival and degranulation in tissues; IL-4/13: act on B cells to induce class switching to IgE; IL-13: increases mucous production from epithelial goblet cells, induces bronchoconstriction in smooth muscle and the vasculature to promote edema). These cytokines also polarize macrophages toward an “alternatively activated” M2 phenotype that sustains tissue remodeling and fibrosis [86]. In the CRS nasal mucosa, chronically inflamed epithelial and endothelial cells express high levels of adhesion molecules such as VCAM-1 and secrete chemokines (e.g., eotaxins, RANTES) that specifically recruit eosinophils and Th2 cells to the tissue [87]. Such adhesion and chemotactic molecules are also upregulated in AR, linking AR to the potential to exacerbate CRS by directing allergic effector cells into the sinuses. In summary, the dysfunctional epithelium in CRS is not just a bystander but a key player that both responds to and sustains inflammation, particularly in allergic (T2) endotypes.

### 6.4. Unifying Feedback Loops and Knowledge Gaps

However, the field still lacks longitudinal, deeply phenotyped cohorts designed to test whether “allergic CRS” constitutes a distinct microbiome-defined entity versus a clinical overlay on broader CRS endotypes. Future research should standardize allergic phenotyping, control for the use of intranasal corticosteroids and antibiotics, and integrate sequencing with functional measurements such as metabolomics and host transcriptomics to move beyond association and identify actionable microbial targets [55,59].

Taken together, the available data support a unifying conceptual model in which epithelial barrier dysfunction and impaired mucociliary clearance are well-supported contributors to a permissive sinonasal niche, while specific microbial triggers (including *S. aureus* enterotoxin exposure) are largely associative and hypothesis-generating in humans. A compromised epithelial barrier and reduced microbial diversity create conditions for certain bacteria (like *S. aureus*) to flourish, whose products then drive T2 inflammation (superantigen-mediated IgE, etc.), which further damages the barrier and favors more dysbiosis. Likewise, an atopic immune milieu can alter antimicrobial defenses (as seen with histatin-5 in AFRS, see below Section 7.1), allowing microbes to persist. This bidirectional relationship underlies many of the “vicious cycles” in CRS pathology.

## 7. Type 2/Allergy-Associated CRS Entities: Clinical Prototypes

### 7.1. Allergic Fungal Rhinosinusitis (AFRS)

Fungal rhinosinusitis represents a spectrum of sinus diseases caused or facilitated by fungi, with clinical manifestations ranging from invasive to non-invasive forms. The host’s immune status is the critical determinant of how fungal sinus disease presents. In immunocompetent patients, fungi typically cause non-invasive disease unless there is an abnormal immune response. The non-invasive forms include simple saprophytic fungal colonization, sinus fungal ball, and AFRS, whereas invasive forms (usually in immunocompromised hosts) include acute fulminant invasive fungal sinusitis, chronic invasive fungal sinusitis, and chronic granulomatous fungal sinusitis [88]. AFRS is now recognized as a distinct subtype of CRS in immunocompetent, atopic individuals.

AFRS is characterized by chronic eosinophilic inflammation of the sinuses associated with hypersensitivity to fungal antigens. Patients with AFRS typically experience nasal polyposis and produce thick, sticky eosinophil-rich mucus that appears as a characteristic “peanut butter” or dark green appearance. Histologically, a hallmark is eosinophilic mucin filled with degranulated eosinophils and Charcot–Leyden crystals. Embedded within this mucin are non-invasive fungal hyphae (the fungi do not invade tissue) [89,90]. AFRS patients demonstrate a type I IgE-mediated hypersensitivity to various fungi. Common culprits include *Alternaria, Bipolaris*, *Curvularia*, *Aspergillus*, and *Fusarium* species [89,90]. Total IgE levels are often markedly elevated in AFRS (much higher than in non-fungal CRSwNP) [3]. AFRS accounts for roughly 5–10% of all CRS cases, and about 6–9% of all fungal sinusitis cases reported in some series [89].

Geographically, AFRS has been reported worldwide and appears more frequently in warm and humid regions (e.g., subtropical climates) [70,91]. The maxillary and ethmoid sinuses are most frequently involved, but AFRS can affect multiple sinuses. CT typically shows heterogeneous sinus opacification accompanied by regions of hyperattenuation attributable to allergic mucin [70,89,91]. Bone remodeling or erosion may occur due to pressure effects from accumulated allergic mucin.

Diagnostically, the Bent and Kuhn criteria are classically used for AFRS. These include: (1) evidence of type I hypersensitivity (history of atopy or positive skin/IgE tests to fungi), (2) nasal polyps, (3) characteristic sinus CT findings (e.g., heterogeneous sinus opacification often with bone expansion), (4) eosinophilic “allergic” mucin without invasive fungal hyphae in sinus tissue, and (5) positive fungal stain or culture from sinus contents obtained during surgery [70,89,91,92]. All criteria should be met to definitively diagnose AFRS. In practice, clinicians should maintain a high suspicion in atopic patients presenting with polyps, elevated IgE levels, and sinus CT scans revealing expansile, heterogeneous opacifications. Confirmation is achieved through histopathological examination of surgical samples, which show allergic mucin containing fungi.

Immunologically, AFRS is characterized by an extreme Th2-biased response to fungi. Patients typically exhibit very high levels of fungal-specific IgE, often directed against multiple fungal antigens [70,93]. Additionally, there is evidence of local IgE production in the sinuses (sinus tissues from AFRS patients can produce fungus-specific IgE independently of systemic circulation) [90]. A growing hypothesis is that AFRS patients have an impaired mucosal barrier and Type 3 immune defense, allowing fungal elements to persist on the mucosal surface and continually stimulating a T2 hypersensitivity response. Consistent with this, studies have found that AFRS sinus tissue has reduced levels of certain antifungal innate defense molecules (e.g., the antimicrobial peptide histatin-5), compared to non-AFRS CRSwNP tissue [94]. Histatin has antifungal properties and its expression is normally upregulated by Type 3 cytokines (IL-17, IL-22) in the epithelium [95]. The decreased levels of histatin and other innate defense mechanisms in AFRS indicate a deficient local Th17-type response, which may fail to effectively control fungal growth. The immune system of the AFRS patient may compensate by eliciting an exaggerated Th2 response, characterized by substantial eosinophil recruitment and IgE production, in an effort to eliminate the fungus [96]. Indeed, the sinonasal epithelium in AFRS appears to be a key orchestrator. Damaged epithelial cells release large amounts of TSLP, IL-33, and IL-25, which in turn activate ILC2s and Th2 cells [97]. AFRS sinus tissue contains abundant ILC2s that express receptors for these alarmins and secrete IL-5, IL-13, and IL-4, correlating with markers of severe disease (tissue eosinophilia, polyps, comorbid asthma, high nasal endoscopy scores, etc.) [98]. Therefore, AFRS serves as a model demonstrating how a microbial trigger (fungi), within a susceptible host immune environment, results in chronic T2-dominant inflammation.

Management of AFRS involves a combination of surgical and pharmacological approaches. First-line therapy typically includes functional endoscopic sinus surgery (ESS) to remove the allergic mucin and polyps and ventilate the sinuses, followed by medication to reduce recurrence. These therapies often include saline sinus irrigations, topical and systemic corticosteroids, antifungal agents (topical or systemic, though evidence is mixed), and allergen immunotherapy in selected cases, aiming to reduce the fungal load and modulate the allergic response [3]. Studies are ongoing to determine whether the biologic medications now approved for CRSwNP (e.g., anti-IgE or anti-IL-5, anti-IL-4Rα and anti-TSLP antibodies) will be effective in AFRS. Early reports have been promising. A recent systematic review indicated that dupilumab, omalizumab, and mepolizumab can significantly improve sinus symptoms, endoscopic scores, and biomarkers (IgE, eosinophils) in patients with refractory AFRS [99]. These biologics targeting T2 inflammation may emerge as valuable adjuncts for severe AFRS, although robust controlled trials are needed. Recent reviews have highlighted the significance of standardized diagnostic criteria and a comprehensive, long-term management strategy for AFRS [100,101].

Multi-kingdom profiling in AFRS suggests a low-diversity, dysbiotic bacterial community often dominated by *S. aureus* alongside a mycobiome enriched for taxa such as *Malassezia*, *Aspergillus* and *Curvularia*, supporting potential cross-kingdom interactions that could reinforce eosinophilic mucin formation and T2 inflammation [56,57,58].

### 7.2. Central Compartment Atopic Disease (CCAD)

Central compartment atopic disease is a newly described subtype of CRS (first described in 2017) that links inhalant allergy with a distinctive anatomic pattern of sinus inflammation. CCAD is characterized by polypoid edema and inflammation confined largely to the central nasal structures, specifically the superior portion of the nasal septum and the adjacent turbinates (middle turbinates and superior turbinates) [102,103]. Unlike typical CRSwNP, which often involves the ethmoid and lateral sinuses diffusely, CCAD shows minimal disease in the lateral paranasal sinuses. On CT imaging, CCAD patients have only mild mucosal thickening in the peripheral sinuses and significantly lower Lund-Mackay scores than other CRSwNP, with the inflammation instead concentrated in the central nasal area [104,105]. The endoscopic findings include edema, polypoid change, and small polyps arising from the dorsal septum and the medial aspects of the turbinates. Polyps or polypoid swelling in CCAD can extend from the posterior septum to the middle turbinates, sometimes touching the middle meatus, but the key is that the central compartment (nasal septum and adjacent turbinates) is primarily affected [106]. Any extension to the sinuses (frontal, maxillary) is secondary to obstruction by the central swelling, rather than primary inflammation of those sinus linings.

CCAD appears to be fundamentally driven by inhalant allergy localized to the nose. It has a very strong association with AR. Studies report that 74–100% of CCAD patients are sensitized to common inhalants [107,108]. Basically, CCAD could be considered an “allergy-driven” form of CRS in which chronic allergic edema in the central nasal airway leads to secondary sinus obstruction. Pathologically, CCAD tissues show a T2 high immune profile with IgE deposition and eosinophils, similar to other atopic nasal polyposis, but with a unique distribution. It is an IgE-mediated process involving the central sinonasal compartment, resulting in phenotypic polypoid edema of the posterior septum and the contiguous surfaces of the middle and superior turbinates [102,103,104]. Unlike diffuse CRSwNP, the inflammation rarely affects the lateral ethmoid labyrinth or sinus cavities unless there is secondary obstruction of the ostia.

An interesting clinical feature of CCAD is that, despite being an atopic and eosinophilic condition, asthma is less frequent in CCAD patients compared to other eosinophilic CRSwNP subtypes. Marcus et al. found only a 17.1% prevalence of asthma in CCAD patients, the lowest among the CRSwNP subgroups they studied [107]. This suggests CCAD might represent a more isolated form of upper airway atopy that does not always extend to the lower airway. Furthermore, CCAD patients in that series had significantly fewer prior sinus surgeries compared to other CRSwNP patients, and they experienced lower rates of polyp recurrence following surgery [109]. This indicates that once the central obstruction is relieved (and with ongoing topical therapy), CCAD tends to have a more favorable and durable outcome than diffuse polyposis, possibly because the disease is anatomically limited and tied to controllable allergic triggers.

CCAD patients often maintain better olfaction in early stages because the olfactory cleft may not be as severely involved until later in the disease. Some cases report near-normal sense of smell initially, which can delay diagnosis because patients (and clinicians) might not suspect “sinusitis” in the absence of anosmia [106]. However, other studies (particularly in Asia) have noted that hyposmia or anosmia can be a predominant symptom of CCAD, suggesting that there may be variations by disease stage or patient population [110]. Interestingly, a study from southern China described a form of CCAD where a significant fraction of patients had coexistent asthma and peripheral eosinophilia despite having negative skin or serum allergy tests [108]. This has raised questions about whether CCAD in different ethnic groups may have varying immunologic profiles (for instance, “local” atopy with negative systemic tests, vs. systemic atopy). Overall, CCAD underscores the concept that localized allergic inflammation in the nose can give rise to a unique CRS phenotype with central polypoid changes, relatively limited sinus disease, and generally favorable post-surgical outcomes when appropriately managed.

Direct microbiome studies focused specifically on CCAD remain scarce. Given the strong association with inhalant allergy and inflammation centered on the middle/superior turbinates and adjacent septum, CCAD represents a compelling model to test whether localized atopic inflammation in the “central compartment” is associated with distinct microbial community states compared with diffuse CRSwNP or CRSsNP. Existing work comparing allergic rhinitis, CRS, and healthy controls supports the broader concept that atopic inflammation can reshape sinonasal bacterial composition and diversity [75,76].

### 7.3. Aspirin-Exacerbated Respiratory Disease (AERD)

Aspirin-exacerbated respiratory disease is a well-defined CRS subtype that illustrates the interplay between chronic sinus inflammation, lower airway disease, and drug hypersensitivity. AERD, also known as Samter’s triad or NSAID-exacerbated respiratory disease (N-ERD), is classically defined by the triad of: (1) severe adult-onset asthma, (2) CRSwNP (usually eosinophilic nasal polyps), and (3) hypersensitivity reactions to aspirin and other cyclooxygenase-1 (COX-1) inhibiting NSAIDs [48]. AERD patients often have persistent, aggressive polypoid sinusitis and asthma that is difficult to control. In terms of epidemiology, AERD is estimated to occur in approximately 7% of all adults with asthma, but in up to 14% of adults with severe asthma, and in about 30% of those who have both asthma and nasal polyps [111,112]. Most patients with AERD have adult-onset disease (commonly presenting in the third to fourth decade of life). It is only slightly more common in females. Interestingly, AERD appears to be quite rare in certain Asian populations (e.g., it is rarely reported in China) [113], suggesting possible genetic or environmental factors influence its prevalence. Approximately half of AERD patients report a history of a preceding viral respiratory infection before the onset of their aspirin sensitivity and asthma worsening [114], although the significance of this observation remains unclear.

From a clinical perspective, the CRSwNP in AERD is especially refractory. Patients typically experience severe nasal congestion, thick discharge, and anosmia from diffuse polyposis. Even after aggressive endoscopic sinus surgery, nasal polyps tend to recur rapidly in AERD patients (sometimes within weeks) unless additional therapies (such as aspirin desensitization or biologics) are employed [115]. Compared to aspirin-tolerant CRSwNP patients, those with AERD require more frequent revision surgeries and long-term systemic corticosteroids to maintain control [116]. In summary, AERD-associated CRS is among the most treatment-resistant forms of CRS.

A defining characteristic of AERD is hypersensitivity to aspirin and NSAIDs. Consuming even small amounts of aspirin or other non-selective COX-1 inhibitors (such as ibuprofen and naproxen) can trigger an immediate reaction in these individuals. Within 30–60 min of NSAID ingestion, AERD patients develop profuse rhinorrhea, nasal congestion, conjunctival injection, bronchospasm with wheezing, and often flushing [48,117]. Many will also have gastrointestinal symptoms (abdominal cramping, nausea) and, in some cases, systemic reactions such as urticarial rash or even aspirin-induced pancreatitis [118]. These reactions are due to the dysregulated arachidonic acid metabolism in AERD, specifically, an overproduction of cysteinyl leukotrienes when COX-1 is inhibited (shunting arachidonic acid away from prostaglandin production into the leukotriene pathway). Because the mechanism specifically involves COX-1 inhibition, COX-2 selective NSAIDs (such as celecoxib) are generally tolerated by AERD patients [119]. Highly selective COX-2 inhibitors cannot fit into the COX-1 enzyme binding site and thus do not trigger the reaction. However, partial COX-2 inhibitors or dual COX inhibitors (such as high-dose meloxicam or older agents like diclofenac) can sometimes cause milder reactions if they have significant COX-1 inhibitory activity [119].

All AERD patients, by definition, have asthma—typically eosinophilic asthma that is often severe and persistent. Conversely, not all CRSwNP patients have asthma, and not all asthmatics have NSAID sensitivity. It has been noted that AR is actually more closely associated with asthma than with nasal polyposis. For example, among CRSwNP patients without asthma, about two-thirds have AR, while in those with comorbid asthma, approximately 81% have AR, and about 84% in AERD [68]. This suggests that the presence of asthma increases the likelihood of an atopic background. In AERD, the asthma is often difficult-to-treat, adult-onset and can show features of fixed airway obstruction. Over half of AERD patients have severe asthma that may not fully reverse with bronchodilators (a sign of airway remodeling) [120,121]. Their asthma and CRS are usually linked (poor control of one often worsens the other).

Pathophysiologically, AERD involves a dysfunctional arachidonic acid metabolism and intense T2 inflammation. There is overexpression of leukotriene C4 synthase in respiratory inflammatory cells, leading to overproduction of cysteinyl leukotrienes (LTC4, LTD4, LTE4), which are potent bronchoconstrictors and drivers of eosinophilic inflammation. AERD patients often have elevated baseline urinary LTE4 and show further LTE4 surges after aspirin challenge. They also typically have high levels of eosinophils in blood and tissue, elevated IL-5 and IL-13 in nasal tissues, and often elevated IgE. Recent research has shown that platelet-leukocyte aggregates and mast cell mediators (such as prostaglandin D2) contribute to the acute aspirin reaction, and that respiratory epithelial cells in AERD may maintain a continuous “inflammatory memory” phenotype even in the absence of triggers [85,122]. Clinically, treatments like aspirin desensitization (daily high-dose aspirin therapy after a supervised desensitization procedure) can markedly improve nasal and asthma symptoms by modulating the arachidonic acid pathways, though this approach requires careful patient selection and carries some risks.

In summary, AERD represents an endotype of CRS characterized by allergic-like inflammation that occurs alongside a distinctive, non-IgE drug hypersensitivity. These patients have persistent sinusitis as part of a systemic inflammatory disorder. The role of AR in AERD is notable (most have atopy as mentioned), but managing AERD often goes beyond standard allergy treatment, involving leukotriene-modifying agents, aspirin desensitization, and now biologic drugs (e.g., anti-IL-5, anti-IgE, anti-IL-4Rα) which have shown efficacy in this difficult population.

Besides intense eosinophilic/T2 inflammation and frequent exposure to *S. aureus* superantigens, new data suggest that N-ERD/AERD might be linked to a microbiota enriched with *Staphylococcus* and depleted of *Corynebacterium*, which correlates with local IL-5 and other T2 biomarkers [51,79].

## 8. Therapeutic Implications: From Biologics to Microbiome Modulation

Emerging therapies for CRS are increasingly targeting these underlying mechanisms (Table 3). For instance, biologic medications that neutralize key T2 cytokines or mediators have shown remarkable efficacy in severe CRSwNP. Monoclonal antibodies such as dupilumab (anti-IL-4Rα blocking IL-4/IL-13 signaling), mepolizumab (anti–IL-5), omalizumab (anti-IgE) and tezepelumab (anti-TSLP) are now approved for use in CRSwNP. Clinical trials and real-world studies demonstrate that these biologics can significantly reduce nasal polyp size, improve sinus symptoms and smell, and decrease the need for systemic steroids or revision surgeries [40,123]. Notably, they are particularly beneficial in patients with co-morbid asthma or AERD, underscoring the importance of endotype-driven therapy. At the same time, there is growing interest in microbiome-modifying treatments. Preliminary clinical trials using topical probiotics have yielded encouraging results. In a small study, intranasal irrigation with the probiotic *Lactococcus lactis* W136 in patients with recalcitrant CRS was safe and showed improvements [124] in sinus symptoms, endoscopic findings, and a shift in the nasal microbiome (reducing pathogenic bacteria and increasing beneficial commensals) [125]. However, a recent systematic review and meta-analysis of probiotic interventions in adults with CRS found that improvements in symptom scores and relapse rates were small and not consistently statistically significant, although safety profiles were generally comparable to placebo [126]. These findings indicate that therapeutically restoring the balance of the sinonasal microbiota, such as through probiotics or bacteriophage therapy targeting staphylococcal biofilms, could become a novel adjunct strategy in CRS management [26]. Additional work suggests that after surgery, commercially available rinses (e.g., xylitol-based formulations) and topical bacteriotherapies can measurably shift sinonasal community structure [127]. Bacteriophage approaches show ex vivo activity against *S. aureus* biofilms derived from CRS patients and may represent a future adjunct for biofilm-driven, recalcitrant disease [128]. More recently, nasal microbiota transplantation has been proposed as an emerging approach to restore community resilience, similar to fecal microbiota transplantation for treating gut diseases [129]. In addition to direct microbiota supplementation, anti-inflammatory treatments used in CRS can also be examined from a microbiome perspective. For instance, a study using before–after 16S rRNA sequencing found that administering 1,8-cineole to CRS patients did not cause significant disruption to gut microbial communities [130]. While such approaches are still experimental, they highlight an important principle: addressing epithelial barrier integrity and the microbial community may be just as crucial as modulating the immune response to achieve long-term control of CRS.

Given that allergic rhinitis frequently coexists with T2-high CRS and may contribute to symptom burden, optimizing rhinitis management (allergen avoidance, intranasal corticosteroids/antihistamines, and allergen immunotherapy in appropriately selected patients) remains a rational adjunctive strategy, although direct disease-modifying effects on established CRS are not well defined [1,131].

Beyond ex vivo data, early-phase clinical experience suggests that intranasal bacteriophage therapy targeting recalcitrant *S. aureus*–positive CRS can be feasible and well tolerated, with preliminary microbiological signals in small cohorts [132].

Notably, CRS-associated dysbiosis may comprise distinct community states enriched for *Streptococcus* and oral-associated anaerobes such as *Prevotella* (phylum *Bacteroidetes*), which correlate with divergent inflammatory programs [43,133]. In parallel, epithelial innate immune sensing pathways can directly couple microbial signals to barrier and mucociliary responses. For example, the bitter taste receptor T2R38 recognizes bacterial quorum-sensing acyl-homoserine lactones and stimulates nitric oxide production, which improves mucociliary clearance and exerts antimicrobial effects [134,135,136]. Clinically, TAS2R38 polymorphisms have been associated with refractory CRS requiring surgery and with variability in postoperative outcomes, supporting a potential role for taste-receptor biology as a biomarker and therapeutic target [137,138]. Cytokines such as oncostatin M have the potential to compromise the integrity of tight junctions and sustain epithelial permeability [139], while epithelial P-glycoprotein (ABCB1) may amplify type 2 cytokine responses in CRS and has been proposed as a candidate target for personalized therapy [140,141]. Finally, coagulation and fibrinolysis imbalance also influence inflammatory remodeling in CRSwNP, with impaired tissue plasminogen activator expression and fibrin buildup mediated by factor XIII-A, as observed in nasal polyps and various inflammatory endotypes [142,143,144,145]. Several additional mechanisms depicted in Figure 1 are still emerging and are included to propose testable hypotheses and potential biomarkers or targets, rather than to suggest uniform evidence support. These pathways are biologically plausible but require replication and longitudinal validation to define their role across CRS endotypes and clinical outcomes.

## 9. Conclusions and Future Directions

Chronic rhinosinusitis (CRS) is best viewed as a heterogeneous inflammatory disorder shaped by interactions between epithelial barrier integrity, sinonasal microbial ecology, and immune programs across distinct phenotypes and endotypes. In many patients, type 2 inflammation co-occurs with barrier impairment and dysbiosis/biofilms, supporting a model in which these domains can reinforce one another, although directionality likely varies by endotype and remains incompletely resolved in humans. Within this review, we use the term “allergic CRS” as a working heuristic descriptor for T2-high CRS with clinically relevant allergic features, rather than as a universally accepted stand-alone disease entity. However, converging clinical prototypes with prominent atopic signatures (e.g., AFRS and CCAD), together with recurrent observations linking allergic status to specific sinonasal microbial patterns in subsets of patients, raise the possibility that “allergic CRS” may represent a reproducible, clinically meaningful subgroup worthy of more explicit recognition and prospective validation. Accordingly, a key near-term priority is to move from descriptive, cross-sectional associations to longitudinal, deeply phenotyped cohorts that harmonize allergic phenotyping (sensitization vs. clinically active allergic rhinitis), tissue biomarkers of type 2 inflammation, and standardized microbiome profiling linked to outcomes such as treatment response and postsurgical recurrence. Such an approach would allow the field to test whether an “allergic CRS” construct improves risk stratification and guides selection of adjunctive allergy-directed and endotype-directed therapies beyond existing CRS frameworks. As a narrative synthesis, this review necessarily reflects the heterogeneity of the current evidence base, but it also defines concrete criteria for the studies required to determine whether T2-high CRS with allergic features or simply “allergic CRS” should evolve from a pragmatic label into a distinct, validated entity.

## Figures and Tables

**Table 1:** Clinically oriented overview of three type 2–enriched CRS phenotypes emphasized in this review. Note: Phenotypes are clinical syndromes, whereas endotypes are biomarker-defined inflammatory mechanisms, and overlap is expected.

Clinical Phenotype (Prototype)	Key Diagnostic Clues/Clinical Context	Typical Endotype Features (Common, Not Exclusive)	Clinical Relevance/Management Cues
**Allergic fungal rhinosinusitis (AFRS)**	AFRS typically presents as CRSwNP with allergic mucin and evidence of fungal hypersensitivity in an atopic context.	AFRS is commonly T2-high/eosinophilic with elevated IgE and prominent type 2-skewed inflammation.	Management is usually multimodal and centers on surgery plus anti-inflammatory control, with attention to comorbid allergic disease.
**Central compartment atopic disease (CCAD)**	CCAD is characterized by inflammation/polypoid changes predominating in the central compartment (e.g., the middle turbinate/olfactory cleft region) and is strongly associated with inhalant allergy.	CCAD frequently aligns with a T2-high pattern, but inflammatory expression may vary across patients and populations.	Allergy evaluation and control are clinically central, with topical therapy and surgery used as indicated by anatomy and extent.
**Aspirin-exacerbated respiratory disease (AERD/N-ERD)**	AERD is a clinical syndrome defined by CRSwNP with asthma and respiratory reactions to COX-1 inhibitors, and it is often associated with severe, recurrent disease.	AERD is commonly T2-high/eosinophilic and may show strong type 2 mediator signatures within polyp tissue.	Recognition of the syndrome guides integrated upper–lower airway care and supports consideration of endotype-directed escalation in appropriate patients.

Note: Phenotypes refer to clinical syndromes, whereas endotypes refer to biomarker-defined inflammatory mechanisms (overlap is expected).

**Table 2:** Representative culture-independent studies informing microbiome–atopy/T2 linkages in CRS. The table prioritizes study population/comparator, allergic definition where applicable, design, and the main microbiome conclusion relevant to an “allergic CRS” framework.

Study (Ref.)	Cohort	Allergy/Atopy Definition	Design	Key Microbiome Conclusion Relevant to Allergic/T2-High CRS
Mahdavinia et al., 2018 [74]	CRS cohort with allergic phenotyping.	Allergic status/AR assessed within CRS.	Cross-sectional culture-independent bacterial profiling.	Allergic status/AR associated with differences in relative abundance and predicted functional pathways (e.g., lower *Corynebacterium* spp. in allergic vs. non-allergic CRS), supporting stratification of CRS by allergic features.
Lal et al., 2017 [75]	Healthy controls vs. AR vs. CRS.	Clinical AR group (definition per study).	Cross-sectional bacterial community profiling.	Group-level differences in composition/diversity across health, AR and CRS, positioning AR as a distinct sinonasal habitat state relevant when interpreting CRS dysbiosis signals.
Gan et al., 2021 [76]	AR vs. CRS vs. controls.	Clinical AR group (definition per study).	Cross-sectional bacterial community profiling.	Differences in diversity and taxonomic composition between AR, CRS and controls, reinforcing that AR should not be treated only as a covariate in CRS microbiome analyses.
Abreu et al., 2012 [49]	Rhinosinusitis-associated vs. healthy sinonasal microbiomes.	N/A.	Cross-sectional 16S profiling with mechanistic modeling.	Reduced community diversity with enrichment of specific taxa (e.g., *Corynebacterium tuberculostearicum*), supporting a commensal-depletion/pathobiont-enrichment dysbiosis framework compatible with barrier–immune hypotheses.
Feazel et al., 2012 [61]	CRS vs. controls; middle meatus swabs during ESS.	N/A.	Cross-sectional culture vs. 16S sequencing comparison.	Culture-independent profiling revealed greater biodiversity than culture and showed altered composition with higher *Staphylococcus aureus* in CRS; diversity correlated with recent antibiotics and asthma, highlighting key confounders for microbiome–atopy inference.
Ramakrishnan et al., 2015 [42]	CRS phenotypes and surgical outcome.	N/A.	Cross-sectional 16S profiling with outcome linkage.	Sinonasal microbiota differed across CRS phenotypes and was associated with postoperative outcomes, supporting microbiome-informed stratification relevant to precision CRS frameworks.
Anderson et al., 2016 [62]	Systematic review of culture-independent CRS microbiome studies.	N/A.	Systematic review.	Marked heterogeneity across cohorts/sampling/analytics with no single taxon consistently linked to CRS, emphasizing the need for standardized design and endotyping (including allergic/T2 markers) in future work.
Cope et al., 2017 [43]	CRS patients with divergent clinical/immune features.	N/A.	Community and functional analyses.	Distinct community states were linked to divergent mucosal immune profiles and clinical consequences, supporting a host–microbe “state” model rather than a single pathogen paradigm.
Wagner Mackenzie et al., 2017 [63]	Meta-analysis/ecological synthesis of CRS vs. controls.	N/A.	Meta-analysis/ecological synthesis of 16S datasets.	Supported a “bacterial community collapse” model with reduced diversity and ecological disruption despite inter-study variability, informing restorative approaches rather than pathogen-only targeting.
Chalermwatanachai et al., 2018 [54]	CRSwNP vs. controls.	N/A.	Cross-sectional 16S profiling.	CRSwNP was characterized by dysbacteriosis of the nasal microbiota, aligning with T2-high polyp-disease frameworks and providing background for atopy-associated subtypes.
Kim et al., 2020 [77]	CRSwNP with eosinophilic inflammation.	N/A.	Cross-sectional profiling and correlations.	Microbial patterns correlated with eosinophilic inflammation, directly linking community structure with T2-relevant tissue inflammation.
Liang et al., 2023 [50]	Eosinophilic CRS vs. comparators.	N/A.	Cross-sectional 16S profiling.	Eosinophilic CRS exhibited distinct microbiome alterations compared with non-eosinophilic disease, supporting endotype–microbiome coupling relevant to allergic/T2-high phenotyping.
Kidoguchi et al., 2023 [78]	Japanese cohort; eosinophilic CRS focus.	N/A.	Cross-sectional middle meatus profiling.	Microbiome differences were described in eosinophilic CRS in a Japanese population, highlighting geographic/ethnic variability and the need for harmonized sampling/confounder control.
Bartosik et al., 2023 [79]	Steroid-free N-ERD/AERD vs. comparator CRS/controls.	N/A.	Cross-sectional profiling in absence of corticosteroids; correlations.	In steroid-free N-ERD, increased staphylococci and reduced corynebacteria correlated with IL-5 and other T2 markers, supporting microbiome–T2 links while addressing corticosteroid confounding.
Connell et al., 2024 [56]	AFRS vs. CRSwNP (non-fungal).	N/A.	Multi-kingdom profiling (full-length 16S and fungal ITS).	AFRS showed lower bacterial diversity and *Staphylococcus aureus* dominance with a mycobiome enriched in *Malassezia/Aspergillus/Curvularia*, supporting a multi-kingdom dysbiotic microenvironment in allergy-associated CRS.

Abbreviations: AERD, aspirin-exacerbated respiratory disease; AFRS, allergic fungal rhinosinusitis; AR, allergic rhinitis; CRS, chronic rhinosinusitis; CRSwNP, chronic rhinosinusitis with nasal polyps; ESS, endoscopic sinus surgery; IL, interleukin; ITS, internal transcribed spacer; N-ERD, NSAID-exacerbated respiratory disease; NSAID, nonsteroidal anti-inflammatory drug; rRNA, ribosomal RNA; T2, type 2.

**Table 3:** Therapeutic strategies for chronic rhinosinusitis (CRS) positioned within the proposed host–epithelial barrier–microbiome “triad,” summarizing for each modality its predominant axis of action (immune, barrier/habitat, and/or microbiome), the overall level of evidence in CRS (high-level), the phenotypes/endotypes most likely to benefit, and key implementation considerations from a microbiome/ecology perspective (including potential effects on microbial habitat and implications for interpreting microbiome studies).

Therapy Class	Primary Target in the Triad (Immune/Barrier/Microbiome)	Evidence Level in CRS (Very High-Level)	Most Relevant Phenotypes/Endotypes	Key Practical Notes/Limitations (Microbiome Perspective)
Intranasal corticosteroids + saline irrigations [3,40]	Immune + barrier (reduce inflammation; improve mucociliary clearance)	Established standard of care	Broad CRS; particularly adjunctive in T2-high disease and atopy-associated phenotypes	May indirectly modulate microbial habitat by improving clearance and reducing inflammatory exudate; confounding factor in microbiome studies (should be documented/standardized).
Endoscopic sinus surgery (ESS) [3,40]	Barrier/habitat “reset” (ventilation, drainage, access for topical treatments)	Established standard of care in selected patients	Recalcitrant CRS; diffuse CRSwNP; AFRS; AERD	Alters local ecology and enables topical therapies; postoperative microbiome trajectories may relate to recurrence risk [43].
Biologics targeting T2 pathways (anti-IL-4Rα, anti-IgE, anti-IL-5, anti-TSLP) [123]	Immune (T2 axis)	High-level evidence (RCTs/real-world in CRSwNP)	T2-high CRSwNP; AERD; severe eosinophilic disease; refractory AFRS (emerging) [99]	Primarily host-directed; may secondarily reshape microbiome by reducing T2 inflammation and epithelial injury; optimal integration with microbiome-directed adjuncts is unknown.
Aspirin desensitization + leukotriene-modifying therapy (AERD) [48,117]	Immune mediator balance (eicosanoid/leukotriene pathways)	Established in AERD specialty care	AERD/N-ERD	Mechanism is not IgE-mediated; requires careful selection/monitoring; may reduce need for repeated antibiotics/steroids, indirectly influencing dysbiosis.
Antifungal-directed strategies (AFRS) [3,100,101]	Microbiome (mycobiome) + immune (reduce fungal antigenic drive)	Variable/heterogeneous evidence	AFRS	Evidence for routine antifungals is mixed; ESS + steroid-centric anti-inflammatory control remains core; multi-kingdom profiling may help identify responders.
Allergen immunotherapy (AIT) and optimized AR control [1,131]	Immune (allergen-specific tolerance) + barrier (reduce chronic allergic edema)	Established in AR; adjunct role in CRS is phenotype-dependent	CCAD; CRS with strong inhalant allergy comorbidity	Likely benefits the “allergic habitat” rather than directly treating dysbiosis; careful phenotyping needed (systemic vs. local allergic rhinitis).
Topical probiotics/bacteriotherapy (e.g., *Lactococcus lactis* W136) [124,125,126,127]	Microbiome + barrier repair (competitive exclusion; epithelial regeneration signals)	Early-phase clinical studies	Refractory CRS (post-surgery cohorts studied)	Promising safety signals; needs larger controlled trials with standardized endpoints and longitudinal microbiome + host readouts [124].
Bacteriophage therapy (anti-*S. aureus* biofilms) [128,132]	Microbiome (pathobiont/biofilm targeting)	Preclinical/ex vivo evidence	Biofilm-associated, recalcitrant CRS where *S. aureus* implicated	Mechanistically attractive for biofilm disease; clinical delivery, resistance, and regulatory pathways remain open questions.
Nasal microbiota transplantation (NMT) concepts [129]	Microbiome (community restoration/resilience)	Conceptual/early translational	Future candidate approach for dysbiosis-dominant states	Requires rigorous donor/recipient selection, safety frameworks, and definition of “healthy” sinonasal communities.

Abbreviations: AERD, aspirin-exacerbated respiratory disease; AIT, allergen immunotherapy; AFRS, allergic fungal rhinosinusitis; AR, allergic rhinitis; CCAD, central compartment atopic disease; CRS, chronic rhinosinusitis; CRSwNP, chronic rhinosinusitis with nasal polyps; ESS, endoscopic sinus surgery; IgE, immunoglobulin E; IL, interleukin; N-ERD, NSAID-exacerbated respiratory disease; NMT, nasal microbiota transplantation; NSAID, nonsteroidal anti-inflammatory drug; RCT, randomized controlled trial; *S. aureus*, *Staphylococcus aureus*; T2, type 2.

## Data Availability

No new data were created or analyzed in this study. Data sharing is not applicable to this article.
